# Nine Cases of Methanogenic Archaea in Refractory Sinusitis, an Emerging Clinical Entity

**DOI:** 10.3389/fpubh.2019.00038

**Published:** 2019-03-04

**Authors:** Elisabeth Sogodogo, Mustapha Fellag, Ahmed Loukil, Vanessa Demonfort Nkamga, Justin Michel, Patrick Dessi, Pierre-Edouard Fournier, Michel Drancourt

**Affiliations:** ^1^Aix Marseille University, IRD, MEPHI, IHU-Méditerranée Infection, Marseille, France; ^2^Assistance Publique–Hôpitaux de Marseille, Service ORL et Chirurgie Cervico-Faciale, Hôpital de la Conception, Marseille, France; ^3^Aix Marseille University, IRD, VITROME, IHU-Méditerranée Infection, Marseille, France

**Keywords:** *Methanobrevibacter oralis*, *Methanobrevibacter smithii*, *Methanobrevibacter massiliense*, sinusitis, genotyping, archaea, methanogen

## Abstract

The authors report the cases of 9 patients eventually diagnosed with methanogenic archaea refractory or recalcitrant chronic rhinosinusitis, a condition known to involve various anaerobic bacteria but in which the role of methanogenic archaea is unknown. The authors retrospectively searched these microorganisms by PCR in surgically-collected sinusal pus specimens from patients diagnosed with refractory sinusitis, defined by the persistance of sinus inflammation and related-symptoms for more than 12 weeks despite appropriate treatment. Of the 116 tested sinus surgical specimens, 12 (10.3%) from 9 patients (six females, three males; aged 20–71 years) were PCR-positive. These specimens were further investigated by fluorescence *in-situ* hybridization, PCR amplicon-sequencing and culture. *Methanobrevibacter smithii* was documented in four patients and *Methanobrevibacter oralis* in another four, one of whom was also culture-positive. They were associated with a mixed flora including Gram-positive and Gram-negative bacteria. In the latter patient, “*Methanobrevibacter massiliense*” was the sole microorganism detected. These results highlight methanogenic archaea as being part of a mixed anaerobic flora involved in refractory sinusitis, and suggest that the treatment of this condition should include an antibiotic active against methanogens, notably a nitroimidazole derivative.

## Introduction

Methanogenic archaea, the only microorganisms able to produce methane, are acknowledged members of the gut microbiota (1). In recent years, they have emerged as opportunistic pathogens and have been isolated in mixed anaerobic floras in a case of muscular abscess (2) and several cases of brain abscess (3, 4). In the latter situation, *Methanobrevibacter smithii* and *Methanobrevibacter oralis* have been identified by metagenomics and sequencing of specific DNA sequences, while *M. oralis* has also been isolated (3). In these cases of methanogen-associated brain abscess, the source of infection has remained hypothetical (4).

Herein, we report a series of nine patients in whom methanogens were documented in refractory maxillary sinusitis. This condition is observed in approximately 20% of patients in whom chronic rhinosinusitis is resistant to standard antibiotic treatments. Also named recalcitrant or hard-to-treat rhinosinusitis, refractory sinusitis has a significant impact on the patients' quality of life (invalidating symptoms, repeated demands for medical care and treatments (5). The presence of methanogenic archaea in refractory sinusitis, which could constitute an underlying condition preceding methanogen-induced brain abscess, is challenging as these microorganisms are resistant to many first-line antibiotics used in sinusitis, including beta-lactams and macrolides (6).

## Case Report

A 62-year-old woman without any underlying disease, presented with a 2-year medical history of refractory, left maxillary sinusitis. Clinical and biological investigations did not find any underlying deficit in humoral and cellular immunity. The patient benefited a left maxillary puncture and routine culture of the pus yielded *Pseudomonas aeruginosa, Staphylococcus aureus, Raoultella ornithinolytica, Streptococcus pseudointermedius*, and *Corynebacterium accolens* (Table 1). In parallel, complementary investigations of the pus specimen yielded *M. oralis* which was documented by microscopic examination using fluorescent *in situ* hybridization (FISH), polymerase chain reaction (PCR) amplification, and sequencing of the methanogenic archaeal 16S rRNA and *mcr*A genes, and culture. The patient was treated by intravenous ceftazidime and netilmycine combined with oral ofloxacin and rifampin; and nasal application of mupirocin. Over the two further years, the patient presented signs and symptoms of refractory sinusitis despite repeated treatments with oral pristinamycin, ciprofloxacin, and amoxicillin-clavulanate. This case prompted the search for methanogens in a series of sinusal pus specimens collected in patients diagnosed with refractory sinusitis.

**Table 1:** Clinical information on the nine patients from whom archaea were detected in sinusitis pus.

**Case**	**Clinical information**						**Microbiology**						
							**Bacteria**		**Methanogens**				
								**Species**	**Species**	**Sequence similarity**	**FISH**	**Culture**	
	**Sex**	**Age (years)**	**Underlying condition**	**Atopy**	**Localization**	**Treatment**	**Specimen number**			**16S rRNA**	**mcrA**		
1	Female	62	None	N	Left maxillary	Ceftazidine + Netilmycin Ofloxacin + rifampin	*1*	*Corynebacterium accolens* *Staphylococcus aureus* *Raoultella ornithinolytica* *Streptococcus pseudointermedius* *Pseudomonas aeruginosa*	*M. oralis*	99%	100%	Positive	Positive
2	Male	64	Undifferentiated carcinoma nasopharyngeal type, cavum	N	Right maxillary	Amoxicillin- clavulanate	*2*	*S. aureusStaphylococcus epidermidisP. aeruginosaPropionibacterium acnes*	*M. smithii*	99%	99%	Negative	Negative
3	Female	56	None	N	Frontal, ethmoidal, and maxillary	Amoxicillin-clavulanate + ciprofloxacin	*3.1*	*S. aureus*	*M. smithii*	99%	99%	Negative	Negative
							*3.2*	*S. aureus*	*M. smithii*	100%	100%	Negative	Negative
							*3.3*	*S. aureus*	*M. smithii*	100%	100%	Negative	Negative
4	Female	64	Maxillary papilloma	N	Right maxillary	NA	*4*	*Klebsiella oxytoca*	*M. smithii*	100%	99%	Negative	Negative
5	Male	20	None	N	Right frontal ethmoidal maxillary	Amoxicillin- clavulanate + ciprofloxacin	*5*	Sterile	“*M. massiliense*”	100%	Negative	Negative	Negative
6	Female	44	Asthma	Y	Left maxillary Right maxillary	Cotrimoxazole	*6.1*	*S. epidermidis* *Corynebacterium propinquum* *Corynebacterium avidum*	*M. oralis*	100%	99%	Positive	Negative
							*6.2*	*S. epidermidis* *C. propinquum*	*M. oralis*	100%	99%	Negative	Negative
7	Male	64	-MALT lymphoma -Sinusal aspergilloma	N	Left frontal ethmoidal	NA	*7*	*P. aeruginosa* *S. epidermidis* *P. acnes*	*M. oralis*	99%	100%	Negative	Negative
8	Female	71	Undifferentiated carcinoma nasopharyngeal type, cavum	N	Right maxillary	Amoxicillin- clavulanate	*8*	*S. epidermidis* *P. acnes*	*M. oralis*	100%	100%	Positive	Negative
9	Female	53	Nasal and sinusal polyposis	Y	Bilateral maxillary	Amoxicillin- clavulanate	*9*	*Propionibacterium granulorum* *S. aureus* *Haemophilus influenzae*	*M. smithii*	100%	NA	Positive	Negative

*The detail on microorganisms detected in each patient is also provided. “N” stands for absence, “Y” for presence. NA, not available*.

This study conforms to the ethical guidelines of the 1975 Declaration of Helsinki and received the approval of the local IHU Méditerranée-Infection Ethics Committee under n°2016–020. Patients' specimens were anonymized. We retrospectively investigated the presence of methanogenic archaea in a collection of 116 sinus surgical specimens from patients with refractory sinusitis diagnosed at Timone public hospital from December 2016 to December 2017 by using PCR-based detection as a screening method. In all patients, surgical drainages of affected sinuses were part as the medical management along medical therapeutic procedures. Pus specimens have been collected in a transport medium Σ-Transwab® (Elitech France, Puteaux, France) or in a sterile pot, and have been preserved at −80°C until further use. They were routinely analyzed according to our laboratory procedures, including the research of bacteria and fungi. Culture of bacteria was performed at 37°C in 5% sheep blood–enriched Columbia agar and PVX agar (bioMérieux, Marcy l'Etoile, France) under aerobic and anaerobic atmosphere, for 48 h. All microbial colonies that grew on agar plates were identified by our systematic matrix-assisted laser desorption-ionization time-of-flight mass spectrometry (MALDI-TOF-MS) screening using a Microflex spectrometer (Bruker Daltonics, Bremen, Germany) (7). In addition, each pus sample was analyzed for the search of methanogenic archaea as described below.

In a first step, we used PCR-based assays to screen for the presence of methanogens in the 116 pus specimens. For each tested specimen, a 250 μL suspension of pus was used for DNA extraction as previously described (8). A sterile water and Transwab® medium was used as a negative control for each batch of DNA extraction. Amplification and sequencing of the 16S rRNA (primers used: SDArch0333aS15, 5′-TCCAGGCCCTACGGG-3′, and SDArch0958aA19, 5′-YCCGGCGTTGAMTCCAATT-3′) and methyl-coenzyme M reducer (*mcrA*) (primers used: mcrAFor, 5′GCTCTACGACCAGATMTGGCTTGG-3′ and mcrARev, 5′- CCGTAGTACGTGAAGTCATCCAGCA-3′) genes were performed as previously described (8). Nucleotide sequences were assembled using Chromas Pro software, version 1.7 (Technelysium Pty Ltd., Tewantin, Australia) and compared to the GenBank database by similarity search using BLASTN (http://www.ncbi.nlm.nih.gov/blast/). Of the 116 tested refractory sinusitis surgical specimens collected from December 2016 to December 2017, PCR-based screening yielded 12 (10.3%) positive for 16S rRNA including 10 (8.6%) that were also positive for the *mcr*A gene specific for methanogenic archaea. PCR-positive specimens were obtained from nine patients including three Caucasian males and six Caucasian females with an age range of 20–71 years (Table 1). Five patients presented a medical history of Ear-Nose-Throat disorder and two patients had a past history of atopy (Table 1). Investigations of lymphocytes, immunoglobulins, and complement in four patients, yielded normal results in three patients (patients n°1, 6, and 7) and found a 1.9G/L lymphopenia and a normal CD4/CD8 ratio in patient 2. Information on the antibiotics that they had received was available for 7 patients. Five of them had received amoxicillin-clavulanate, including 2 who had also been treated with ciprofloxacin (Table 1). None of the 7 patients had received a nitroimidazole derivative, fucidic acid or lovastatin. Sequencing confirmed the presence of *M. oralis* in patients 1, 6, 7, and 8 and of *M. smithii* in patients 2, 3, 4, and 9, all of whom had polymicrobial cultures. In case-5, sequencing identified “*Methanobrevibacter massiliense*” while routine culture remained sterile for bacteria (Table 1).

**Figure 1:**
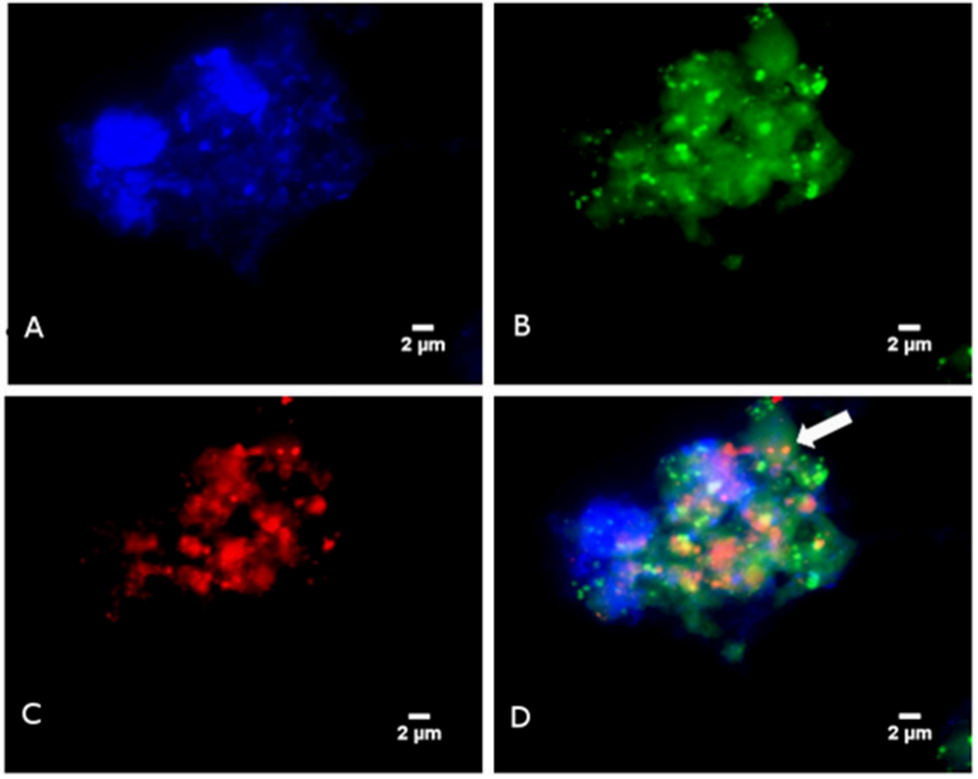
Detection of *Methanobrevibacter oralis* in a sinusitis pus specimen using FISH **(A)** Blue color represents DAPI fluorescence staining any DNA. **(B)** Green color represents EUB338 fluorescence staining bacterial DNA. **(C)** Red color represents ARC915 fluorescence staining the archaeal DNA **(D)** Fluorescence *in situ* hybridization combining EUB338 fluorescence, ARC915 fluorescence and DAPI: the arrow points to archaea.

In a second step, FISH was used to microscopically observed methanogens. Briefly, FISH was carried-out using the Arch915 probe labeled with Alexa fluor-546 (specific for archaeal 16S rRNA gene) and the EUB338 probe labeled with Alexa fluor-488 (specific for eubacterial 16S rRNA gene), as previously described (9) The universal DNA stain 4′,6-diamidine-2′-phenylindole dihydrochloride (DAPI) was added. Microscopy was performed with an epifluorescence microscope DMI 6000 (Leica, Nanterre, France). FISH revealed the presence of archaea in four PCR-positive specimens but none of the PCR-negative samples. The morphology of archaea detected by FISH was suggestive of *M. smithii* in one and *M. oralis* in three patients (Figure 1).

In a third step, culture was used to confirm the viability of methanogens detected by PCR. Therefore, or each PCR-positive specimen, 250 μL of pus was cultured for methanogenic archaea in a Hungate tube containing 5 mL anaerobe methanogenic SAB medium complemented with 100 mg/L imipenem, 100 mg/L vancomycin, and 50 mg/L amphotericin B and incubated in an atmosphere made of 80% H_2_ + 20% CO_2_ at 2 bar pressure at 37°C, with agitation, for 12 weeks, as previously described (10, 11). Sterile PBS was used as a negative culture control. None of the negative control tubes mock-inoculated with PBS yielded evidence for methane production over 12 weeks. Likewise, no methane was detected in any of the tubes inoculated with pus samples except for the tube inoculated with pus from patient 1 in which methane production indicated that a growing methanogen was detected after 12 week incubation. This isolate was identified as *M. oralis* based on a 99% 16S rRNA and a 100% *mcrA* gene sequence similarity with *M. oralis* strain ZR^T^ (GenBank accession NR_104878.1 and NZ_LWMU00000000.1, respectively).

## Discussion

We report the first series of methanogenic archaea-associated sinus infections in patients presenting with refractory sinusitis. Our retrospective PCR-based analysis of 116 sinus specimens sampled over 1 year yielded an estimated prevalence of 10% of methanogens among surgically-sampled patients. Twelve specimens were positive for *Methanobrevibacter* species in nine unrelated patients. In these patients, we documented in six cases *M. smithii*, in five cases *M. oralis*, and in one case “*M. massiliense*” (9, 12) as part of the pus flora. The negativity of negative controls introduced in FISH, culture and PCR-based assays further eliminated in-laboratory contamination by fastidious methanogens. The recovery of *M. oralis* in sinusitis pus could have been anticipated as *M. oralis* had previously been detected in the dental plaque and related periodontitis lesions (13, 14), but the recovery of *M. smithii* in 6 of the 12 sinusitis pus specimens that we investigated, is more surprising. Indeed, *M. smithii* is an acknowledged gut methanogen (15) but it has rarely been detected, and has been cultivated only once from the oral cavity (15). Likewise, “*M. massiliense*” has primarily been detected in ancient dental plaque specimens (9) and has only be recently cultured from a periodontitis specimen (12).

However, we acknowledge the fact that the small number of positive patients in this study did not enable us to draw any conclusion on the exact role and distribution of methanogenic archaeae in refractory sinusitis. Indeed, present study was not properly designed to address the important question of the relative role of methanogens in chronic sinusitis as it was a retrospective study which eventually included only one-culture-positive patient. This patient has not received antibiotics known to be effective against *M. oralis*. Altogether, these observations did not allow for an accurate interpretation of the relative role of methanogens in the pathology. In addition, their isolation is an insufficient criterion to prove their pathogenicity. Refractory sinusitis is acknowledged as a condition linked to mixed infections and the present cases confirm *M. oralis, M. smithii*, and “*M. massiliense*” as three additional microorganisms linked to this disease (16). Moreover, the presence of methanogenic archaea in sinuses fills an anatomical gap between methanogen-associated brain abscess (2) and oral pathologies linked to *M. oralis* including severe periodontitis and peri-implantitis (14, 17) and endodontic infections (18).

Methanogenic archaea are naturally resistant to many antibiotics, notably beta-lactams and macrolides that are frequently used as first-line antibiotics in sinusitis (6, 19, 20). In addition, quinolones are inconstantly active on archaea (6). In our study, 5 of the 7 patients for whom the treatment was known had received amoxicillin-clavulanate and 3 had received quinolones. Methanogenic archae are *in vitro* susceptible only to fusidic acid, nitroimidazole derivatives, and lovastatin (19, 20). In the present study, none of the 7 patients had been treated with any of these drugs, which may, at least in part, explain why the treatment used was inefficient.

We are now able to routinely cultivate aerobically methanogens in anaerobic infections and have already reported that *M. smithii* can be responsible for muscular abscess (2). The present report may prompt doctors and clinical microbiologists to document microbiologically additional cases of sinusitis and sinusal abscess. Indeed, in case of documented methanogenic sinusal infections, the antibiotic management should be reconsidered to include drugs active on these microorganisms, notably fusidic acid, ornidazole and its derivates and lovastatin (19, 20).

## Author Contributions

JM and PD took care of the patients. P-EF and MD performed microbiological analysis. ES, MF, AL, and VN performed methanogen analyses. All the authors drafted and approved the final version of the manuscript.

### Conflict of Interest Statement

All authors have submitted the ICMJE Form for Disclosure of Potential Conflicts of Interest. Conflicts that the editors consider relevant to the content of the manuscript have been disclosed.
